# Utilization of optimal antenatal care, institutional delivery, and associated factors in Northwest Ethiopia

**DOI:** 10.1038/s41598-023-28044-x

**Published:** 2023-01-19

**Authors:** Tesfahun Hailemariam, Asmamaw Atnafu, Lemma Derseh Gezie, Binyam Tilahun

**Affiliations:** 1 Department of Health Informatics, Institute of Public Health, College of Medicine and Health Sciences, University of Gondar, Gondar, Ethiopia; 2 Department of Health System and Policy, Institute of Public Health, College of Medicine and Health Sciences, University of Gondar, Gondar, Ethiopia; 3 Department of Epidemiology and Biostatistics, Institute of Public Health, College of Medicine and Health Sciences, University of Gondar, Gondar, Ethiopia

**Keywords:** Health care, Medical research

## Abstract

Optimal antenatal care visits (ANC4+) and institutional delivery are essentials to save lives of the women and the baby during pregnancy and delivery. Though focused antenatal care visits and institutional delivery is recommended by World Health Organization, Ethiopia has sub-optimal antenatal care and lagged facility delivery. A community-based cross-sectional study was conducted among 811 lactating women in Northwest Ethiopia. Multivariable logistic regression analysis was performed using 95% confidence level and *p* < 0.05. The prevalence of optimal antenatal care visits and institutional delivery were 39.6% (95%CI: 36.2–43) and 62.6% (95%CI: 59.2–66), respectively. Maternal education (AOR = 2.05; 95%CI: 1.14, 3.69), home visiting by health extension workers (AOR = 1.57; 95%CI: 1.01, 2.29), and early antenatal care booking (AOR = 11.92; 95%CI: 8.22, 17.31) were significant predictors of optimal antenatal care. Exposure to mass media (AOR = 1.65; 95% CI: 1.02, 2.65); intended pregnancy(AOR = 1.68; 95%CI:1.12, 3.63); parity of one (AO = 3.46; 95% CI: 1.73, 6.89); 1–3 antenatal care visits (AOR = 2.17; 95% CI: 1.29, 3.63); and ANC4 + (AOR = 3.57; 95% CI: 2.07, 6.14); history of pregnancy-related complications(AOR = 1.63; 95%CI: 1.04, 2.57), and access to transportation to reach a health facility(AOR = 1.58; 95%CI: 1.00, 2.45) were significant predictors of institutional delivery. Addressing the modifiable factors identified in this study could improve optimal antenatal care visit and institutional delivery.

## Introduction

Globally, countries are investing huge efforts to achieve the pledged annual rate of maternal mortality reduction by 7.5% from 2016 to 2030^1^. Though high maternal mortality countries had an ambitious plan to reduce preventable maternal mortality, it is the experience and rates of change in a few countries that provided inspiration and guidance on how to achieve the necessary acceleration to reduce preventable maternal deaths^1^.

Statistics show that more than 298, 800 women die annually due to pregnancy and childbirth complications. The vast majority (94%) of these deaths occur in low and middle-income countries^2^. From the global figure, approximately 14,000 maternal deaths occur each year in Ethiopia, and the lifetime risk of maternal mortality is 1:55 for a 15-year-old woman^3^. The current evidence shows that four women die from every 1000 live births in Ethiopia, which is the highest maternal death in the world^4^.

Of all maternal deaths reported, 75% would have been averted if mothers had received proper maternal healthcare services^5^. Evidence showed that preventable maternal deaths are mainly attributed to limited access to health services^6^, a dearth of skilled healthcare providers^6^ and a low level of maternal health service utilization^4^.

Women’s completion of the recommended Antenatal Care (ANC) visit is a rallying call to protect pregnancy, childbirth, and postpartum-related complications and deaths^7^. In developing countries, only 40% of women attend at least four ANC visits during pregnancy^1^. Similarly, the average level of birth attended by healthcare providers was 59% in developing countries and 44% in SSA^8^. World Health Organization (WHO) recommended at least four ANC for every pregnant woman^9^. However, the current recommendation increases the number of ANC contacts to eight. This, thus, help provide a comprehensive platform for health promotion, disease prevention, screening, diagnosis, and management and betterment of maternal and perinatal outcomes, while the four contacts are still an unfinished agenda in Ethiopia^10^.

Although remarkable progresses and improvements were observed in maternal mortality reduction and maternal health, the completion of optimal ANC visits and institutional delivery is low and has continued as a challenge for maternal health programs in Ethiopia^11^. According to the national report^12^, only 32% of women receive optimal antenatal care visits and less than half (48%) of women give birth in a health facility, which is much lower as compared to the target set by the government of Ethiopia to achieve 95% of at least four ANC utilization^12^ and 90% of institutional delivery^13^.

There is a shortage of data on the level and factors associated with attending at least four antenatal care visits during pregnancy and institutional delivery. Accordingly, we considered a community-based study in order to assess the level of optimal antenatal care visit utilization, institutional delivery and associated factors.

Maternal health service utilization could be affected by access to health services, governmental commitment, and socio-cultural factors. The findings of this study could inform planners and policy makers to design and implement an intervention to improve maternal health program. Therefore, this study aimed to identify utilization and factors associated with optimal antenatal care visits and institutional delivery in rural Northwest Ethiopia.

## Methods

### Study area and period

This study was conducted in two districts of the Central Gondar Zone, Northwest Ethiopia. The districts are located 658 km from Addis Ababa, the capital city of Ethiopia. There were 16 health centers and 88 health posts in the districts. The total population was 524, 907 (female = 260,879 and male = 264,028) at the time of the survey, of which 122,303 and 16,745 were women in the reproductive age group and surviving infants, respectively. The study period of this study was from October to November, 2020.

### Study design

A community-based cross-sectional study was conducted.

### Source population

All lactating women who gave birth within the past 12-months and living in the study districts were the target population.

### Study population

A sample of lactating women who gave birth within the past 12-months who were permanent residents of the selected kebeles were the study population.

### Sample size determination

Both the single population proportion and the double population proportion formulas were considered in estimating different sample sizes. For the single population proportion formula, the assumptions used were: prevalence of ANC attendance^7^, prevalence of completion of four or more antenatal care visits^7^, the proportion of mothers who received delivery service from health care providers after completing antenatal care four and above and the proportion of women retained in the continuum of maternal care^7^, considering 95% CI, the margin of error = 5%, design effect of 2 and 10% non-response rate. For double population proportion formulas, the sample size was computed using the STAT-CALC program of Epi-info version 7.0. software using the following assumptions: 5% level of significance (two-sided), 80% power, and a 1:1 ratio of exposed to non-exposed and odds ratio. Factors such as being a model household in the community^14^, distance to reach a health facility^15^, and antenatal care visits^16^ were considered predictor variables. The design effect of 2 and 10% non-response rate were used during different sample size estimations. Of the different sample sizes estimated, the maximum sample size, 811, was obtained from a single population formula and considered in the study.

### Sampling procedure

A multi-stage cluster sampling technique was employed to reach study participants. Wogera and Gondar zuriya districts were selected randomly among the six transformation woredas in the central Gondar zone. Specifically, the primary sampling units were kebeles with the respective health centers while the secondary sampling units were women who gave birth during the past 12 months. The health centers (or the respective kebeles) were selected with a simple random sampling technique. To select sample participants, a sampling frame of mothers was developed using a list of eligible women from the community health information system family folder(pouch). Then the sampling interval was obtained by dividing the source population (8393) by the estimated total sample size (811). The first study participant (the 2nd mother) was selected by a simple random sampling technique from the first sampling interval, and all other mothers were selected systematically by taking every 10th mother in the frame. When two or more women were found at a single household level, one of them was selected randomly and included in the study. Seriously ill or women who were unable to speak were excluded from the study. For those women who were selected for an interview and were not available during data collection, we waited for them for three to five days to get them. If they were not still accessible, they would be considered non-respondent.

### Variables and measurement

#### Outcome variables

The outcome variables of this study were optimal antenatal care (ANC4 +), and institutional delivery. The optimal antenatal care was classified as “yes” if the women had optimal antenatal care. i.e., attending four or more antenatal care visits during pregnancy, or “no” if they had less than four antenatal care visits. Similarly, women who gave birth at health facilities (health centers or hospitals) were categorized as “yes” or “no” if otherwise.

#### Explanatory variables

Explanatory variables were the age of the respondents, education status of respondents, education status of partner, intendedness of the current pregnancy, being a member of community-based health insurance, mass-media exposure, health extension workers' home visits during pregnancy, being a member of Women’s Development Army, early ANC booking, pregnancy-related complications, planned pregnancy, frequency of ANC visits, parity, access to transportation, and household wealth index. For wealth index assessment, principal component analysis was employed to reduce data that measure the same construct together, and the data were recoded into binary variables. The wealth quantile was categorized into five categories: poorest, poor, middle, richer, and richest.

#### Data collection procedures and tools

Interviewer-administered questionnaire was used to collect the household data. The tool was prepared in English after a thorough literature review and translated into the local language (Amharic) and then finally returned to English. Since experts’ views were sought for the psychometric properties of the face and content validity, experts were invited to review the relevance of each question in the tool. The tool was refined according to the experts view and piloted out of the study setting and validated before data collection. The proportions of the rated item content validity index and scale content validity index were 0.98 and 0.81, respectively. Six data collectors and two supervisors were recruited for data collection and supervision. The data were collected using interviewer administered questionnaire via face-to-face interview.

#### Data quality assurance

A two-day training was given to data collectors and supervisors regarding the objective of the study, procedures of the data collection, data collection tools and handling of the data. During the data collection period, daily supervision was conducted by the supervisors.

#### Data management and statistical analysis

Data were entered into EpiData 4.02 and then exported to STATA 14 software for statistical analysis. Principal Component Analysis (PCA) was constructed to assess household wealth status, and the items were recoded into two categories (0 = no, 1 = yes) to reduce the amount of data that measure the same construct. Twenty-one items composed of household productive and non-productive assets and utilities were entered for analysis. The frequencies of the assets greater than 95% and less than 5% were excluded from the analysis as it would not help to identify the richer or poorer category. The scale reliability coefficient and Kaiser–Meyer–Olkin measure of sampling adequacy were employed to assess the satisfaction of the assumptions for PCA. The wealth quantile was categorized into five categories: poorest, poor, middle, richer, and richest. To identify factors associated with response variables, bivariable logistic regression analysis was fitted. Likewise, the model fitness was checked using the Hosmer and Lemeshow goodness of fitness test. The correlation of independent variables was checked by using the Variance Inflation Factor (VIF), in which VIF > 10 indicates substantial multicollinearity, and there was no multicollinearity effect among predictor variables. Descriptive statistics such as frequency and cross-tabulation were computed. In the bivariable logistic regression model, a *p* < 0.25 was used to recruit candidate variables for multivariable model adjustment, and the selected variables were entered sequentially by using backward stepwise regression. An association between outcome variables and explanatory variables was presented using an adjusted OR with a 95% confidence interval. A significance level of *p* < 0.05 was considered to declare the level of association with women’s optimal antenatal care visits and institutional delivery.

### Ethical considerations

Study approval and ethical clearance were obtained from the University of Gondar ethical review board (R.NO. V/P/RCS/05/2020). A formal letter of approval was taken from Amhara national regional health state bureau and central Gondar zonal health department. Written informed consent was taken from participants and their parents and assent obtained from the minor/participant. They were informed about the objective, importance of the study, procedure and duration, risk and discomfort, benefits of participating in the study, confidentiality, and the right to refuse or withdraw during data collection. After obtaining the relevant information, participants were counselled on the benefits of attending maternal healthcare services and the consequences of missing maternal health care services. All methods were carried out in accordance with relevant guidelines and regulations.

## Results

### Socio-demographic characteristics of the study participants

A total of 811 women participated in this study, with a 100% response rate. The median age of the participants was 28 years (± 10), with an interquartile range of 23–33 years. The proportion of women who had attended secondary education and above was 96 (11.8%). The proportion of women was nearly equal across the wealth quantiles of the household level, with the poorest 163 (20.1%), poorer 162 (20.0%), middle 163 (20.1%), richer 161 (19.8%), and the richest 162 (20.0%). According to this study, 142 (17.5%) of women reported that they had access to transportation (Table 1).

**Table 1:** Sociodemographic demographic characteristics of delivered mothers in Northwest Ethiopia, 2020.

Variables	Categories	Frequency (N, %)
Age group of the respondents (years)	15–24	227 (27.9)
	25–34	394 (48.6)
	35 and above	190 (23.4)
Occupation of the respondents	Housewife	801 (98.8)
	Others (merchant, employee and daily laborer)	10 (1.2)
Maternal education	No formal education	500 (61.7)
	Primary education	215 (26.5)
	Secondary and above	96 (11.8)
Paternal education	No formal education	523 (64.5)
	Primary education	205 (25.3)
	Secondary and above	83 (10.2)
Household wealth status	Poorest	163 (20.1)
	Poorer	162 (20.0)
	Middle	163 (20.1)
	Richer	161 (19.8)
	Richest	162 (20.0)
Transportation access	Yes	142 (17.5)
	No	669 (82.5)

Source of information and community participation of the study participants.

This study showed that the proportion of women who had received home visits by HEWs (Health Extension Workers) during pregnancy was found to be 361 (44.5%). Women who reported that they had access to mass media exposure were 140 (17.3%). Our study revealed that, of the total 811 mothers, 394 (48.6%) delivered mothers were members of community-based health insurance and 260 (32.1%) of the mothers were members of the women’s development army (Table 2).

**Table 2:** Source of information about antenatal care visits and community participation related variables among delivered mothers in Northwest Ethiopia, 2020.

Variables	Categories	Frequency (N, %)
Home visiting by health extension workers	Yes	361 (44.5)
	No	450 (55.5)
Exposure to mass media (TV or radio)	Yes	140 (17.3)
	No	671 (82.7)
Member of community-based health insurance	Yes	394 (48.6)
	No	417 (51.4)
Member of women development army	Yes	260 (32.1)
	No	551 (67.9)
Getting health education during pregnancy	Yes	493 (60.8)
	No	318 (39.2)

### Reproductive health characteristics of the study participants

In this study, 358 (50.9%) women had attended antenatal care visits within 16 weeks of gestational age, and 321 (45.6%) had completed four and above antenatal care visits. Of the women who had received at least one ANC visit, 358 (50.9%) had attended the first ANC visits before 16 weeks of gestational age, whereas 346 (49.1%) had attended the first antenatal care visits after 16 weeks of gestational age. Our study found that 177 (21.8%) of the women were parity one, whereas 276 (34.1) of the participants had given birth to five or above. Of all the participants, 642 (79.2%) had the intention to their recent pregnancies. According to our findings, 127 (15.7%) of the women had exposure to pregnancy-related complications (Table 3).

**Table3:** Antenatal care visit status and obstetric history among delivered mothers in Northwest Ethiopia, 2020.

Variables	Categories	Frequency (N, %)
Have you attended ANC for recent pregnancy (n = 811)	Yes	704 (86.8)
	No	107 (13.2)
Number of ANC visits (n = 704)	One time	33 (4.7)
	Two times	97 (13.8)
	Three times	253 (35.9)
	Four and above	321 (45.6)
In which trimester have you attended an ANC visit? (n = 704)	Within 16 weeks	358 (50.9)
	16 up to 28 weeks	268 (38.0)
	28 up to 34 weeks	68 (9.7)
	34 up to 40 weeks	10 (1.4)
The current pregnancy was intended (n = 811)	Yes	642 (79.2)
	No	169 (20.8)
The current pregnancy was planned (n = 811)	Yes	635 (78.3)
	No	176 (21.7)
Parity (n = 811)	1	177 (21.8)
	2–4	358 (44.1)
	> = 5	276 (34.1)
Pregnancy-related complications (n = 811)	Yes	127 (15.7)
	No	684 (84.3)

### Utilization of optimal antenatal care visits and institutional delivery

**Figure 1:**
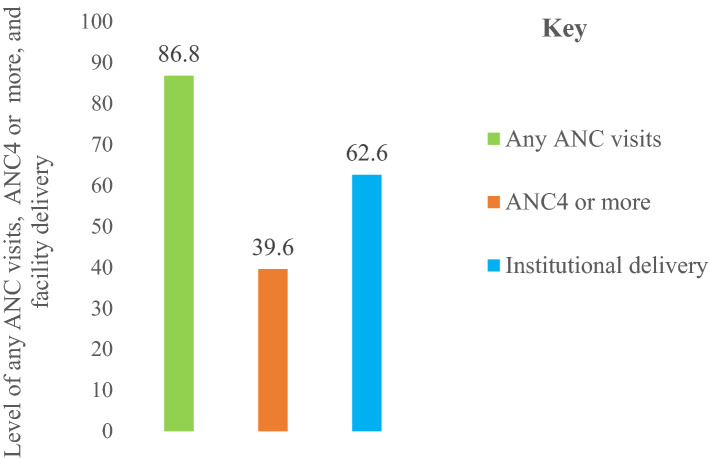
Any ANC visits, ANC4 or more, and institutional delivery in Northwest Ethiopia, 2020.

The proportion of mothers who had received ANC visits from skilled healthcare providers (doctors, health officers, nurses, and midwives) was 86.8% (95% CI: 84.3–89.1). Of the mothers who had received ANC visits during pregnancy, 39.6% (95% CI: 36.2–43) of them continued to complete optimal ANC visits. Our study found that 62.6% (95% CI: 59.2–66) of the mothers gave birth at health facilities (hospitals or health centers) (Fig. 1).

### Main reasons for antenatal care visit non-attendance

**Figure 2:**
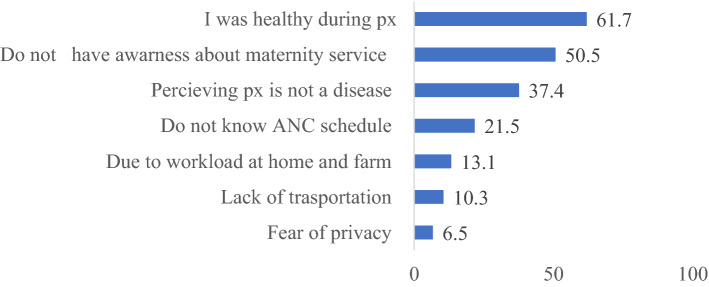
Reasons for antenatal care visits non-attendance in Northwest Ethiopia, 2020.

The main reasons for non-attendance of ANC visits among 107 respondents who did not attend ANC visits during pregnancy were: being healthy during pregnancy period 66 (61.7%), not having awareness about the importance of receiving antenatal care from health care providers 54 (50.5%), women perception that pregnancy was not a disease 40 (37.4%), and fear of privacy 7 (6.5%) (Fig. 2).

### Main reasons for preferring home delivery

**Figure 3:**
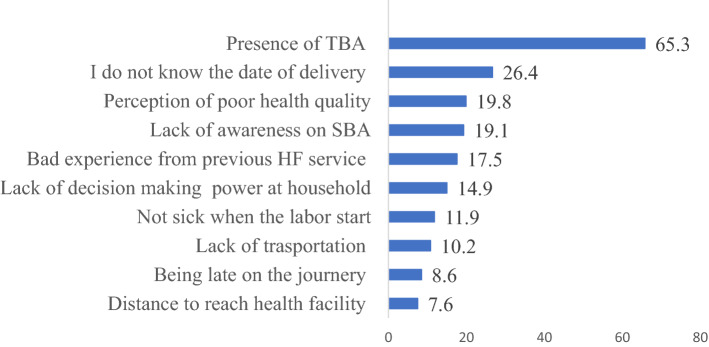
Reasons for preferring home delivery in Northwest Ethiopia.

Among 303 women who gave birth at home, 198 (65.3%) reported that they preferred home delivery because of presence Traditional Birth Attendants (TBA). Those women who did not know the date of delivery and perceived that they could not get quality healthcare were 80 (26.4%) and 60 (19.8%), respectively. This study found that about 23(7.6%) of women gave birth at home due to distance to reach health facilities (Fig. 3).

### Factors associated with optimal antenatal care visit

Women who attended secondary and above-education were 2.05 times (AOR = 2.05; 95%CI: 1.14, 3.69) more likely to complete optimal antenatal care as compared to women who did not attend formal education. This study revealed that women who had received home visits by health extension workers were 1.57 times (AOR = 1.57; 95%CI: 1.01, 2.29) more likely to attend optimal ANC than their counterparts. Our study also found that women who had attended ANC booking before 16 weeks of gestational age were 11.92 times (AOR = 11.92; 95%CI: 8.22, 17.31) more likely to utilize optimal antenatal care as compared to women who had attended antenatal care visits after 16 weeks of gestational age (Table 4).

**Table 4:** Factors affecting optimal antenatal care in a rural Northwest Ethiopia, 2020.

Variables	Category	Optimal ANC		COR (95%CI)	AOR (95%CI)
		Yes	No		
Age group of the respondents (years)	15–24	79	148	0.76 (0.52, 1.14)	0.82 (0.46,1.46)
	25–34	164	230	1.02 (0.72,1.46)	0.90 (0.57,1.43)
	35 and above	78	112	1	1
Women education	No formal education	187	313	1	1
	Primary education	77	138	0.93 (0.67, 1.30)	0.92 (0.57, 1.47)
	Secondary and above	57	39	2.45 (1.57, 3.82)	2.05 (1.14, 3.69)*
Home visits by HEWs	Yes	178	183	2.09 (1.57, 2.78)	1.57(1.01, 2.29)*
	No	143	307	1	1
Planed pregnancy	Yes	275	360	2.12 (1.49, 3.13)	1.14 (0.49, 2.67)
	No	46	130	1	1
Member of health insurance	Yes	178	216	1.58 (1.19, 2.09)	1.17(0.81, 1.69)
	No	143	274	1	
Member of women’s development army	Yes	122	138	1.56 (1.16, 2.11)	1.07(0.72, 1.59)
	No	199	352	1	
Intended pregnancy	Yes	279	363	2.32 (1.59, 3.41)	0.78 (0.33, 1.85)
	No	42	127	1	1
Pregnancy related complications	Yes	61	66	1.51 (1.03, 2.21)	1.49 (0.92, 2.43)
	No	260	424	1	1
Early ANC booking	Yes	259	99	11.98 (8.37, 17.17)	11.92 (8.22, 17.31)**
	No	62	284	1	1

*Significant at *p* < 0.05.

**Significant at *p* < 0.00.

### Factors associated with institutional delivery

According to our study, women who had exposure to mass media were 1.65 times (AOR = 1.65; 95%CI: 1.02, 2.65) more likely to attend institutional delivery as compared to their counterparts. The odds of giving birth in a health facility were 1.68 times higher (AOR = 1.68; 95% CI: 1.12, 3.63) in women who had the intendedness of the current pregnancy as compared to women who had no intention to their pregnancies. Women who had a birth order of 1 were 3.46 times (AOR = 3.46; 95%CI: 1.73, 6.89) more likely to give birth at a health facility than women who had a birth order of ≥ 5**.** Our study revealed that women who had 1–3 ANC visits were 2.17 times (AOR = 2.17; 95%CI: 1.29, 3.63) more likely to give birth at a health facility as compared to women who had not attended any antenatal care visits during pregnancy. Similarly, women who had ANC4 + visits were 3.57 times (AOR = 3.57; 95% CI: 2.07, 6.14) more likely to give birth in a health facility than women who did not attend any antenatal care visit. Besides, this study identified that having pregnancy-related complications was significantly associated with institutional delivery (AOR = 1.63; 95% CI: 1.04, 2.57). Our study also found that women who had access to transportation to reach health facilities were 1.58 times (AOR = 1.58; 95%CI: 1.00, 2.45) more likely to attend institutional delivery than women who had no access to transportation to reach health facilities (Table 5).

**Table 5:** Factors affecting institutional delivery in a rural Northwest Ethiopia, 2020.

Variables	Category	Institutional delivery		COR (95%CI)	AOR (95%CI)
		Yes	No		
Age group of the respondents (years)	15–24	161	66	1.85 (1.24, 2.78)	0.75 (0.40, 1.39)
	25–34	239	155	1.17 (0.82, 1.66)	1.14 (0.75, 1.72)
	35 and above	108	82	1	1
Women education	No formal education	279	221	1	1
	Primary education	149	66	1.79 (1.27, 2.51)	1.36 (0.88, 2.08)
	Secondary and above	80	16	3.96 (2.25, 6.97)	1.76 (0.86, 3.59)
Husband education	No formal education	303	220	1	1
	Primary education	134	71	0.32 (0.02, 0.65)	0.97 (0.66, 1.43)
	Secondary and above	71	12	1.46 (0.82, 2.09)	1.97 (0.92, 4.23)
Mass-media exposure	Yes	108	32	2.29 (1.49, 3.49)	1.65 (1.02, 2.65)*
	No	400	271	1	1
Access to transport service	Yes	103	39	1.72 (1.15, 2.57)	1.58 (1.00, 2.45)*
	No	405	264	1	1
Intended pregnancy	Yes	432	210	2.52 (1.78, 3.55)	1.68(1.12, 3.63)*
	No	76	93	1	1
Parity	1	148	29	3.59 (2.26, 5.72)	3.46 (1.73, 6.89)***
	2–4	198	160	0.87 (0.63, 1.19)	0.75 (0.50, 1.12)
	> = 5	162	114	1	1
Pregnancy related complications	Yes	91	36	1.62 (1.07, 2.45)	1.63 (1.04, 2.57)*
	No	417	267	1	1
Frequency of ANC visits	No visit	40	67	1	1
	1–3 visit(s)	232	151	2.57 (1.65, 4.00)	2.17 (1.29, 3.63)**
	> = 4 visits	236	85	4.65 (2.93, 7.39)	3.57 (2.07, 6.14)***
Household wealth status	Poorest	83	80	1	1
	Poorer	103	59	1.68 (1.08, 2.62)	1.49 (0.92, 2.34)
	Middle	104	59	1.69 (1.09, 2.65)	1.52 (0.94, 2.46)
	Richer	97	64	1.46 (0.94, 2.27)	1.09 (0.67, 1.77)
	Richest	121	41	2.85 (1.78, 4.55)	1.21 (0.68, 2.15)

*Significant at *p* < 0.05; **significant at *p* < 0.01; ***significant at *p* < 0.00.

## Discussion

The finding of this study suggests that the prevalence of optimal antenatal care was two in five and institutional delivery was three in five in Northwest Ethiopia. It also confirmed that maternal education, health extension workers’ home visiting, and initiation of ANC visits before 16 weeks of gestational age were predictors of optimal antenatal care**,** whereas, exposure to mass media, access to transportation, intended pregnancy, parity of one, history of pregnancy-related complications, and frequency of antenatal care visits were predictors of institutional delivery.

WHO suggested every pregnant woman should attend a minimum of four antenatal care visits^9^ and give birth at a health facility with the assistance of a skilled healthcare attendant^4^. Our study found that the level of optimal antenatal care was in consonance with study conducted in Eastern Hararghe 38.3%^17^. However, it was lower as compared to findings of studies conducted in Tigrai Region 63.9%^15^ and Dembecha district 80%^18^ in Ethiopia and abroad in Nigeria 54%^19^. The probable reason for differences could be due to variations in the availability of quality health service provision, and lack of transportation access to reaching health facilities.

This study revealed that optimal ANC was better as compared to findings of previous studies done in Nekmet 32.1%^20^ in Ethiopia and elsewhere in Kenya 32%^21^. The reason for this variation could be the emphasis given by the government of Ethiopia to improve maternal health service utilization considering that it was one of the exempted services in Ethiopia^22^.

In this study, women’s education was significantly associated with optimal antenatal care. This finding is consistent with studies conducted in Ethiopia^23^ and Tanzania^24^. The possible justification might be that educated women could be aware of the benefits of attending antenatal care visits. The other reason could be that educated women might have fewer economic constraints to reach health facilities and prepare transportation access by themselves without waiting for their husbands to accompany them with some financial support^25^. Furthermore, education provides a woman some autonomy in decision-making during reproductive healthcare seeking. Thu**s,** they might not be influenced by waiting their husbands, partners, family, or relatives to decide on their behalf, which are the common sociocultural factors affecting women with no formal education^26^. Moreover, education is a proxy indicator that enables women to be aware of danger signs and pregnancy-related complications^27^.

Women who had received home visits by health extension workers were 1.57 times more likely to attend optimal antenatal care compared to their counterparts. Our finding was consistent with the finding of study conducted in Ethiopia^28^. The possible justification could be that health extension workers' daily engagement in the provision of health services at the household and community level might influence the health-seeking behavior of pregnant women. Health extension workers in Ethiopia are commonly tasked with the provision of maternal health services such as health education and communication, identification of pregnant women, and creation of demand for health services in the community^29^. Findings from a previous study indicated that community mobilization using health extension workers had brought a considerable enhancement in maternal health programs^30^.

Moreover, the odds of optimal antenatal care visits were 11.92 times higher among women who had started ANC visits before 16 weeks of gestational age than their counterparts. The evidence revealed in our study is supported by studies conducted in Ethiopia^7^. The possible justification could be the fact that during early ANC initiation, women might get knowledge about the importance of Focused Antenatal Care (FANC) components, danger signs, and early identification and treatment of pregnancy-related complications^31^. A study showed that early initiation of ANC makes women have the recommended number of antenatal care visits^32^. Besides, Ethiopia has been implementing the FANC service for years, and the service is currently reinforced in health sector development programs^22^ considering maternal healthcare utilization as one of the exempted services. Moreover, the content of antenatal care might enable women to have sufficient ANC visits^24^. Providing quality antenatal care services allows women to complete the recommended number of antenatal care visits^33^. Conversely, women who have initiated antenatal care visits lately could miss an opportunity to receive the intended benefits of FANC components, which could harm the mother and unborn baby^34^. Early initiation of ANC is, thus, a critical indicator to retain women on the optimal antenatal care visit.

Utilization of institutional delivery in this study agreed with findings of studies conducted in Oromia region 60.3%^35^ in Ethiopia, and abroad in Ghana 60.5%^36^. Nonetheless, institutional delivery in this study is better as compared to findings of studies conducted in Sekela district 12.1%^37^ in Ethiopia, and abroad in Nigeria 37%^19^. The reason for the increment could be the commitment of the Ethiopian government and its stakeholders’ engagement in the Home Delivery Free (HDF) program to improve maternal health and service delivery^38^. The other possible justification for the differences might be the availability of community health service programs in concentration on maternal health service utilization using health extension workers (frontline healthcare providers at primary healthcare units) and WDA (women development army). The WDA is a newly emerged platform to network women into one to five groups that enable women to discuss and share their pregnancy-related experiences^39^. Therefore, this might have been the cause for the increased institutional delivery in the current study. Moreover, the reason for the increased maternal health service utilization in the study area could be that the two districts in which this study was conducted were transformation districts in the region for their better performance in health system indicators.

However, the proportion of the current study is still low according to the national plan envisioned to target 90% of institutional delivery^13^. The possible reason might be the fact that the current study was conducted after the implementation of strong monitoring and evaluation strategies of the maternal health programs at the national level^40^. Likewise, our finding was lower as compared to findings of studies conducted in other parts of Ethiopia: Debre Berhan 80.2%^41^, Mana district 86.4%^42^, and elsewhere in Ghana 77.89%^43^. These disparities could be attributed to socio-cultural status, economic status, access to health care, quality of care provision, and awareness differences among the study participants^23^

Our study found that mass-media exposure had a positive association with institutional delivery. This finding is in agreement with findings of studies conducted in Ethiopia^31^ and Malawi^44^. The possible justification might be the fact that health information delivered through television and or the radio could have a pivotal role in shaping women’s health seeking behavior. The available evidence indicated that women who had access to health information were more aware of the benefits of continued maternal healthcare utilization, danger signs, and pregnancy-related complications than their counterparts^45^. A study in Pakistan indicated that women with weekly exposure to mass media gave birth in health facilities as compared to women who were less frequently exposed to mass-media^46^. Moreover, Ethiopian broadcasting corporation has been announcing the benefits of receiving healthcare services from skilled healthcare providers for years through television and radio (the most widespread and accessible mass media in rural areas), as maternal health service utilization advocacy is one of the strategies to enable women to use maternal health service.

The odds of giving birth in a health facility were 1.68 higher in women who had intended the current pregnancy as compared to their counterparts. This finding is corroborated by findings in Ethiopia^27^, and Bangladesh^47^. The possible justification could be that, on one hand, women with an intended pregnancy were more likely to be emotionally, psychologically, and financially prepared for the demands of delivery service. On the other hand, women with unintended pregnancies could be reluctant to seek maternity care for themselves and their newborns. Some may prefer miscarriage because of emotional and social disregard for their unintended pregnancies^48^. Moreover, women who intended to be pregnant were more likely to utilize maternal healthcare services as compared to those women who did not intend their pregnancies^45^.

The likelihood of giving birth at a health facility among women who had a birth order for their first pregnancy was 3.46 times more likely compared to women who had five or more children. This finding is consistent with studies conducted in Ethiopia^49^ and elsewhere in Cambodia^50^. The possible justification could be that women having their first child are more likely to be fearful of pregnancy and intend to have their babies delivered in a health facility than women having their second or third child^51^. A study showed that women perceive that the first pregnancy could bring more risks than subsequent pregnancies, and they become less attentive when the number of pregnancies exceed**s** a certain limit^52^. Furthermore, women who had a history of giving birth safely would feel they are healthy and do not intend to seek delivery service from health facilities^53^. In addition, evidence indicate**s** that women who have many children are less likely to give birth in a healthy institution^54^. Therefore, community health programs should consider community awareness creation to improve institutional delivery among women with multipara.

The likelihood of women attending institutional delivery was 2.17 and 3.57 times greater among women who attended 1–3, and 4 + ANC visits, respectively, than women who had not received any ANC visit during pregnancy. Our finding is in concord with findings of studies conducted in Ethiopia^55^ and abroad in Ghana^43^. This finding could be justified by the fact that the goal-oriented components of FANC received during ANC visits might enable women to adhere to maternity care follow-up. The other justification might be that women at early antenatal care visits receive counseling from healthcare providers about the place of delivery. Besides, they have a chance to get early identification and treatment of pregnancy-related complications that in turn could enable them to have an institutional delivery.

Furthermore, women who faced any medical or obstetric condition during their pregnancies were 1.63 times more likely to give birth in a health facility as compared to their counterparts. The findings of our study were in line with the findings of studies conducted in Ethiopia^56^, and elsewhere in Bangladesh^57^. The probable reason for the similarity might be that women who have identified medical or pregnancy-related complications might be motivated to seek healthcare^57^. Furthermore, the advice from healthcare providers could influence them to give birth at health facilities. It is known that in most instances, maternal deaths occur as a result of obstetric conditions^5^. Our study suggests that maternal health programs should focus on health education about pregnancy-related complications during antenatal care visits and the importance of giving birth at a health facility.

Finally, our study found that, on one hand, women who had access to transportation to reach a health facility were more likely to give birth in a health facility as compared to their counterparts. On the other hand, mothers who could not get easy transport service to travel from their home to the nearest health facility gave birth at home. This finding is similar to findings of previous study done in Ethiopia^56^, wherein lack of access to health facilities was reported as a barrier to a place of delivery. The possible explanation could be the fact that most of the delivered mothers may not have awareness about the due date of delivery and when the labor starts. Likewise, they may not easily get access to transportation to reach health facilities. Lack of transportation access is one of the main factors negatively impacting healthcare service utilization in Ethiopia^58^. It is a fact that reaching health facilities is a challenge. Accordingly, lack of road access, difficult topography, long distances, lack of transportation, and scattered settlements are the main barriers to health service utilization in Sub-Saharan Africa, where Ethiopia is located^59^. Strengthening access to health facilities reduces delays from getting appropriate care and enhances maternal health service utilization.

## Limitations of the study

Recall bias is possible in the current study since we included women who had been breastfeeding for the last 12 months preceding the survey. As women might not remember previous events other than the recent events, the study might be affected by it. Nevertheless, we attempted to specify questions related to the service given during antenatal and natal by probing what was given and how it was given. Finally, we had not considered community and facility-level factors in the current study which could have impact on the individual factors associated with this study. Moreover, due to the nature of the study design employed in the current study, we could not address the cultural factors that could strongly affect maternal healthcare utilization and can be explored by qualitative study.

## Conclusions

The prevalence of optimal antenatal care visits and institutional delivery were found to be low, i.e., approximately three in five women continued to utilize optimal antenatal care, and two in five gave birth at a health institution. Maternal education, home visiting by health extension workers, and early ANC booking were predictors of optimal antenatal care visits, whereas mass-media exposure, intended pregnancy, parity, frequency of antenatal care visits, having medical or pregnancy-related conditions, and access to transportation to reach health facilities were predictors of institutional delivery. The existing health system should consider improving optimal antenatal care visits and facility delivery by considering pregnant women’s education. This could be realized via adult education programs on the benefits of getting maternal healthcare from healthcare providers, strengthening of health extension programs, and improvement of infrastructures such as roads, and transportation access for every pregnant woman to improve maternal health program and service utilization. Moreover, antenatal care visit should be advocated to improve facility delivery.

## Data Availability

The data used and/or analyzed are with correspondence author and available upon reasonable request.

## Abbreviations

ANC: Antenatal care
WHO: World Health Organization
PCA: Principal component analysis
VIF: Variance inflation factor
HF: Health facility
TBA: Traditional birth attendants
FANC: Focused antenatal care
HDF: Home delivery free
WDA: Women development army
HF: Health facility
Px: Pregnancy
SBA: Skilled birth attendants

## References

[1] Alkema L, Chou D, Hogan D, Zhang S, Moller A-B, Gemmill A (2016). Global, regional, and national levels and trends in maternal mortality between 1990 and 2015, with scenario-based projections to 2030: A systematic analysis by the UN Maternal Mortality Estimation Inter-Agency Group. The Lancet.

[2] Organization WH. Trends in maternal mortality 2000 to 2017: Estimates by WHO, UNICEF, UNFPA, World Bank Group and the United Nations Population Division (2019).

[3] WHO U, UNFPA, World Bank Group and the United Nations Population Division. Geneva: World Health Organization. Trends in maternal mortality 2000 to 2017: Estimates 2019. Licence: CC BY-NC-SA 3.0 IGO.

[4] CSACE I. Ethiopia Demographic and Health Survey 2016. Addis Ababa, Ethiopia, and Rockville, Maryland, USA: CSA and ICF (2016).

[5] Say L, Chou D, Gemmill A, Tunçalp Ö, Moller A-B, Daniels J (2014). Global causes of maternal death: A WHO systematic analysis. Lancet Glob. Health.

[6] Bazile J, Rigodon J, Berman L, Boulanger VM, Maistrellis E, Kausiwa P (2015). Intergenerational impacts of maternal mortality: Qualitative findings from rural Malawi. Reprod. Health.

[7] Emiru AA, Alene GD, Debelew GT (2020). Women’s retention on the continuum of maternal care pathway in west Gojjam zone, Ethiopia: Multilevel analysis. BMC Pregnancy Childbirth.

[8] Organization WH. Strategies towards ending preventable maternal mortality (EPMM) (2015).

[9] Organization WH. WHO recommendations on antenatal care for a positive pregnancy experience: World Health Organization (2016). 28079998

[10] Yehualashet DE, Seboka BT, Tesfa GA, Mamo TT, Seid E (2022). Determinants of optimal antenatal care visit among pregnant women in Ethiopia: A multilevel analysis of Ethiopian mini demographic health survey 2019 data. Reprod. Health.

[11] Shiferaw S, Spigt M, Tekie M, Abdullah M, Fantahun M, Dinant G-J (2016). The effects of a locally developed mHealth intervention on delivery and postnatal care utilization; A prospective controlled evaluation among health Centres in Ethiopia. PLoS ONE.

[12] Mini E, Demographic E. health survey 2019: Key indicators report. The DHS Program ICF (2019).

[13] Health FDRoEMo. Health Sector Transformation Plan: 2015/16–2019/20. Federal Ministry of Health Addis Ababa, Ethiopia (2015).

[14] Medhanyie A, Spigt M, Kifle Y, Schaay N, Sanders D, Blanco R (2012). The role of health extension workers in improving utilization of maternal health services in rural areas in Ethiopia: A cross sectional study. BMC Health Serv. Res..

[15] Ftwi M, Gebretsadik GG-E, Berhe H, Haftu M, Gebremariam G, Tesfau YB (2020). Coverage of completion of four ANC visits based on recommended time schedule in Northern Ethiopia: A community-based cross-sectional study design. PLoS ONE.

[16] Shudura, E., Yoseph, A. & Tamiso, A. Utilization and predictors of maternal health care services among women of reproductive age in Hawassa University health and demographic surveillance system site, South Ethiopia: A Cross-Sectional Study. Advances in Public Health. 2020 (2020).

[17] Zelalem Ayele, D., Belayihun, B., Teji, K. & Admassu Ayana, D. Factors affecting utilization of maternal health Care Services in Kombolcha District, eastern Hararghe zone, Oromia regional state, Eastern Ethiopia. International scholarly research notices. 2014 (2014). 10.1155/2014/917058 PMC4897107 27437510

[18] Gedefaw M, Muche B, Aychiluhem M (2014). Current status of antenatal care utilization in the context of data conflict: The case of Dembecha District, Northwest Ethiopia. Open J. Epidemiol..

[19] Dahiru T, Oche OM (2015). Determinants of antenatal care, institutional delivery and postnatal care services utilization in Nigeria. Pan. Afr. Med. J..

[20] Woyessa AH, Ahmed TH (2019). Assessment of focused antenatal care utilization and associated factors in Western Oromia, Nekemte, Ethiopia. BMC. Res. Notes.

[21] Chorongo, D. *et al.* Factors influencing the utilization of focused antenatal care services in Malindi and Magarini sub-counties of Kilifi county, Kenya. *Pan. Afr. Med. J.* **26**, 25(Suppl 2):14. 10.11604/pamj.supp.2016.25.2.10520 (2016). 10.11604/pamj.supp.2016.25.2.10520 PMC5390059 28439338

[22] Health FM (2010). Management Protocol on Selected Obstetrics Topics.

[23] Ayalew TW, Nigatu AM (2018). Focused antenatal care utilization and associated factors in Debre Tabor Town, northwest Ethiopia, 2017. BMC. Res. Notes.

[24] Bayou YT, Mashalla YS, Thupayagale-Tshweneagae G (2016). The adequacy of antenatal care services among slum residents in Addis Ababa, Ethiopia. BMC Pregnancy Childbirth.

[25] Abera A, Asseffa NA, Obssa MS, Balla ET, Koyira MM (2019). Utilization of focused antenatal care service and associated factors among women in Southern Ethiopia. Int. J. Adv. Med. Health Res..

[26] Darteh EKM, Dickson KS, Doku DT (2019). Women’s reproductive health decision-making: A multi-country analysis of demographic and health surveys in sub-Saharan Africa. PLoS ONE.

[27] Shitie, A., Assefa, N., Dhressa, M. & Dilnessa, T. Completion and Factors Associated with Maternity Continuum of Care among Mothers Who Gave Birth in the Last One Year in Enemay District, Northwest Ethiopia. *J. Pregn.* **2020**, 9. Article ID 7019676. 10.1155/2020/7019676 (2020). 10.1155/2020/7019676 PMC7481927 32953177

[28] Afework MF, Admassu K, Mekonnen A, Hagos S, Asegid M, Ahmed S (2014). Effect of an innovative community based health program on maternal health service utilization in north and south central Ethiopia: A community based cross sectional study. Reprod. Health.

[29] Wakabi W. Extension workers drive Ethiopia's primary health care. *Lancet*. **372**(9642), 880. 10.1016/s0140-6736(08)61381-1 (2008). 10.1016/s0140-6736(08)61381-1 18795419

[30] Bogale A, Mekonnen W (2017). Family planning use and its determinants among pastoralist communities of Ethiopia. Soc Sci..

[31] Fseha, B. & Gebremariam, G. Focused Antinatal Care Service Utilization and Associated Factors Among Pregnant Women in Shire, Tigray, Ethiopia. *Biomed. J. Sci. & Tech. Res. Biomed. Res. Network+, LLC.* **15**(2), 11308–11314 (2019).

[32] Beeckman K, Louckx F, Putman K (2010). Determinants of the number of antenatal visits in a metropolitan region. BMC Public Health.

[33] Muchie KF (2017). Quality of antenatal care services and completion of four or more antenatal care visits in Ethiopia: A finding based on a demographic and health survey. BMC Pregnancy Childbirth.

[34] Horn F, Sabova L, Pinterova E, Hornova J, Trnka J (2014). Prevention of neural tube defects by folic acid-awareness among women of childbearing age in Slovakia. Bratisl. Lek. Listy.

[35] Shigute T, Tejineh S, Tadesse L (2017). Institutional delivery service utilization and associated factors among women of child bearing age at Boset Woreda, Oromia regional state, central Ethiopia. J. Women’s Health Care.

[36] Amoakoh-Coleman M, Ansah EK, Agyepong IA, Grobbee DE, Kayode GA, Klipstein-Grobusch K (2015). Predictors of skilled attendance at delivery among antenatal clinic attendants in Ghana: A cross-sectional study of population data. BMJ Open.

[37] Teferra AS, Alemu FM, Woldeyohannes SM (2012). Institutional delivery service utilization and associated factors among mothers who gave birth in the last 12 months in Sekela District, North West of Ethiopia: A community-based cross sectional study. BMC Pregnancy Childbirth.

[38] Kuruvilla S, Bustreo F, Kuo T, Mishra C, Taylor K, Fogstad H (2016). The Global strategy for women’s, children’s and adolescents’ health (2016–2030): A roadmap based on evidence and country experience. Bull. World Health Organ..

[39] Alemayehu M, Belachew T, Tilahun T (2012). Factors associated with utilization of long acting and permanent contraceptive methods among married women of reproductive age in Mekelle town, Tigray region, north Ethiopia. BMC Pregnancy Childbirth.

[40] Canavan ME, Brault MA, Tatek D, Burssa D, Teshome A, Linnander E (2017). Maternal and neonatal services in Ethiopia: Measuring and improving quality. Bull. World Health Organ..

[41] Limenih D, Deyesa A, Berhane E (2016). Assessing the Magnitude of Institutional Delivery Service Utilization and Associated Factors among Mothers in Debre Berhan, Ethiopia. J. Preg. Child. Health..

[42] Yoseph M, Abebe SM, Mekonnen FA, Sisay M, Gonete KA (2020). Institutional delivery services utilization and its determinant factors among women who gave birth in the past 24 months in Southwest Ethiopia. BMC Health Serv. Res..

[43] Kumbeni MT, Apanga PA (2021). Institutional delivery and associated factors among women in Ghana: Findings from a 2017–2018 multiple indicator cluster survey. Int. Health.

[44] Wang Y, Etowa J, Ghose B, Tang S, Ji L, Huang R (2021). Association between mass media use and maternal healthcare service utilisation in Malawi. J. Multidiscip. Healthc..

[45] Dutamo Z, Assefa N, Egata G (2015). Maternal health care use among married women in Hossaina, Ethiopia. BMC Health Serv. Res..

[46] Agha S, Carton TW (2011). Determinants of institutional delivery in rural Jhang, Pakistan. Int. J. Equity Health..

[47] Khan MN, Harris ML, Loxton D (2021). Does unintended pregnancy have an impact on skilled delivery care use in Bangladesh? A nationally representative cross-sectional study using Demography and Health Survey data. J. Biosoc. Sci..

[48] Tesfaye G, Chojenta C, Smith R, Loxton D (2019). Predisposing, enabling and need factors associated with skilled delivery care utilization among reproductive-aged women in Kersa district, Eastern Ethiopia. Reprod. Health.

[49] Kifle D, Azale T, Gelaw YA, Melsew YA (2017). Maternal health care service seeking behaviors and associated factors among women in rural Haramaya District, Eastern Ethiopia: A triangulated community-based cross-sectional study. Reprod. Health.

[50] Wang W, Hong R (2015). Levels and determinants of continuum of care for maternal and newborn health in Cambodia-evidence from a population-based survey. BMC Pregnancy Childbirth.

[51] Toohill J, Fenwick J, Gamble J, Creedy DK (2014). Prevalence of childbirth fear in an Australian sample of pregnant women. BMC Pregnancy Childbirth.

[52] Worku AG, Yalew AW, Afework MF (2013). Factors affecting utilization of skilled maternal care in Northwest Ethiopia: A multilevel analysis. BMC Int. Health Hum. Rights.

[53] Ononokpono, D. N. & Odimegwu, C. O. Determinants of Maternal Health Care Utilization in Nigeria: A multilevel approach. *Pan. Afr. Med. J.* **17** Suppl 1(Suppl 1):2. 10.11694/pamj.supp.2014.17.1.3596. (2014). 10.11694/pamj.supp.2014.17.1.3596 PMC3958146 24643545

[54] Gashaye A, Kibret GD, Bazezew Y, Mengist B (2021). Factors affecting institutional delivery in Ethiopia: A multi-level analysis. Int. J. Afr. Nurs. Sci..

[55] Nigusie A, Azale T, Yitayal M (2020). Institutional delivery service utilization and associated factors in Ethiopia: A systematic review and META-analysis. BMC Pregnancy Childbirth.

[56] Alemi Kebede KH, Teklehaymanot AN (2016). Factors associated with institutional delivery service utilization in Ethiopia. Int. J. Women’s Health.

[57] Ameyaw EK, Ahinkorah BO, Seidu A-A (2020). Does knowledge of pregnancy complications influence health facility delivery? Analysis of 2014 Bangladesh Demographic and Health Survey. PLoS ONE.

[58] Health FDRoEMo. A Roadmap for Optimizing the Ethiopian Health Extension Program: 2020–2035. Federal Ministry of Health Addis Ababa, Ethiopia (2020).

[59] Fisseha G, Berhane Y, Worku A, Terefe W (2017). Distance from health facility and mothers’ perception of quality related to skilled delivery service utilization in northern Ethiopia. Int. J. Women’s Health.

